# Supplementary figures and images for: A framework for smartphone-enabled, patient-generated health data analysis
Source: PeerJ. 2016 Aug 2;4:e2284. doi: 10.7717/peerj.2284 (PMC4975026; doi:10.7717/peerj.2284)

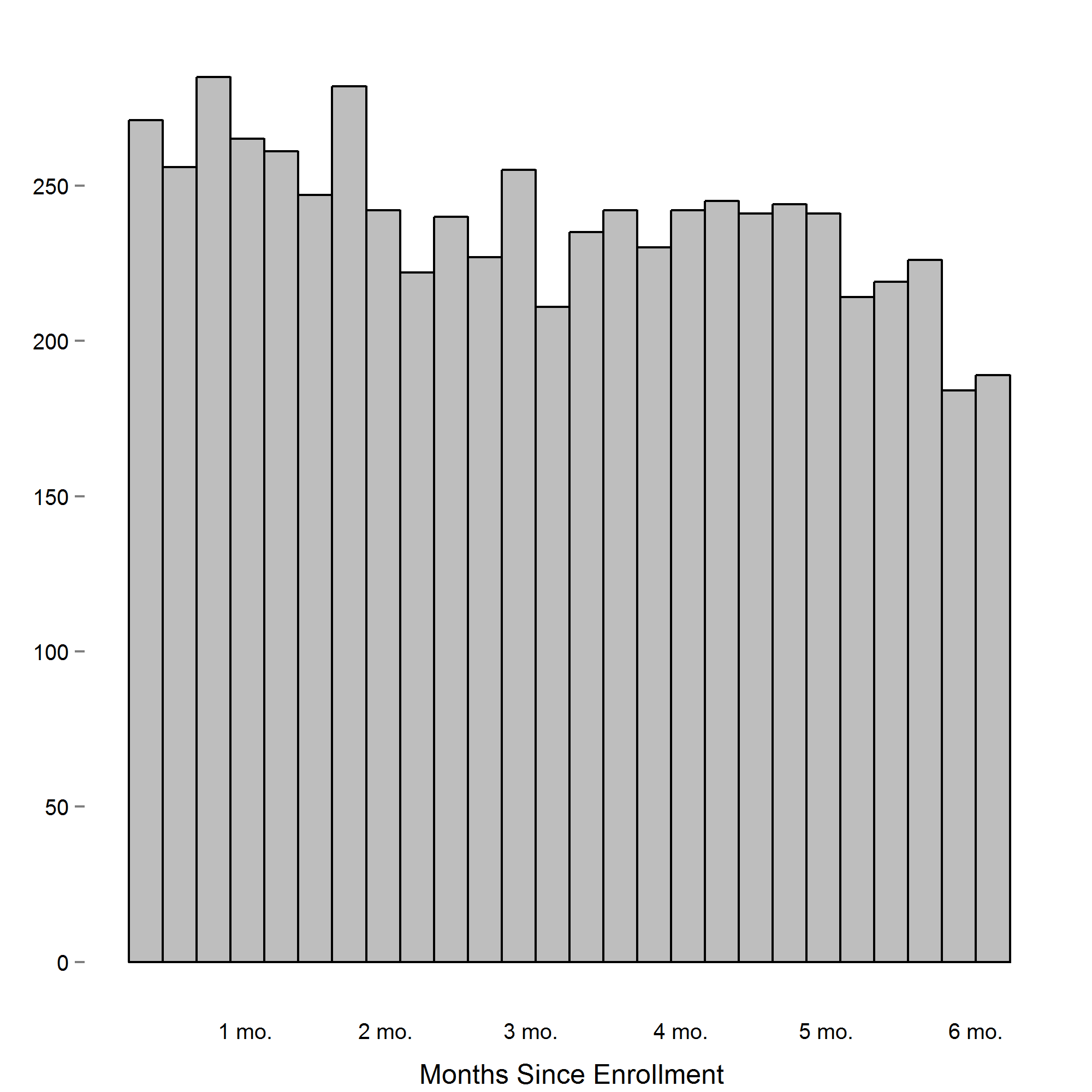

Supplement: Supplemental Information 3 [file peerj-04-2284-s003.png]

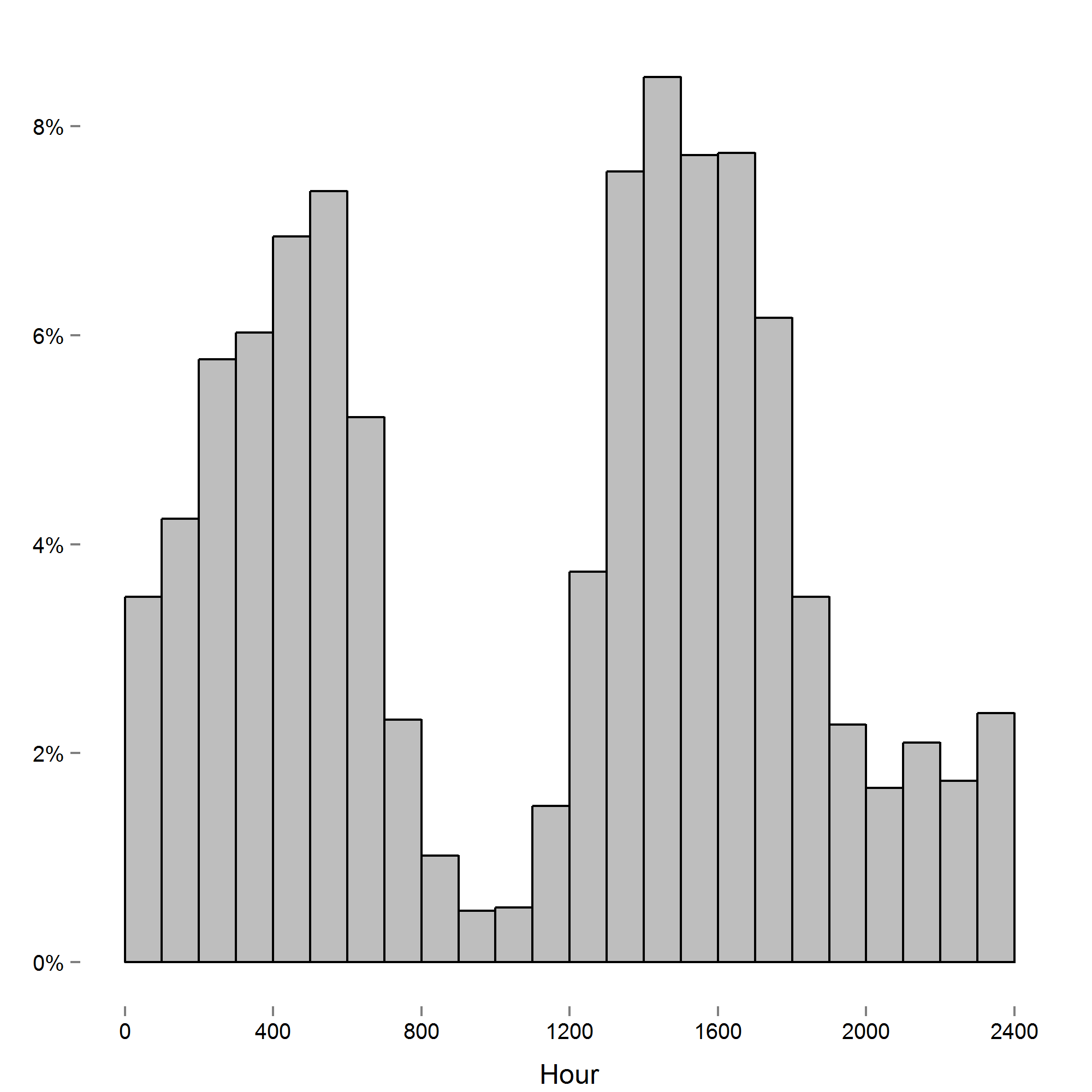

Supplement: Supplemental Information 4 [file peerj-04-2284-s004.png]

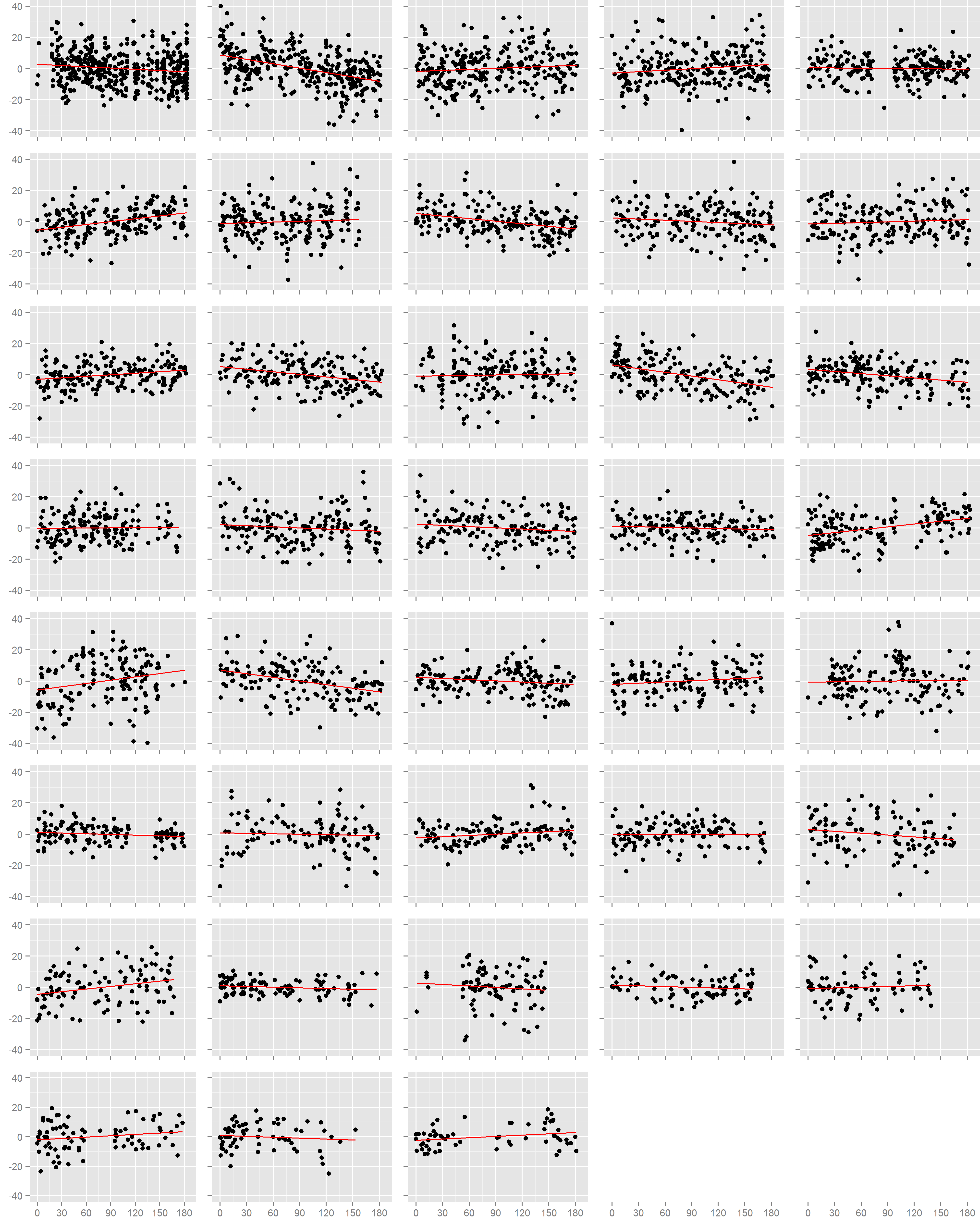

Supplement: Supplemental Information 5 — Each box is one study individual. Points are arranged along the x-axis which represents the time in days from the beginning of the study, and along the y-axis which represents the normalized diastolic blood pressure reading recorded at that time. The red line is the least squares regression line. Individuals are ordered left to right, top to bottom according to the number of readings recorded. [file peerj-04-2284-s005.png]

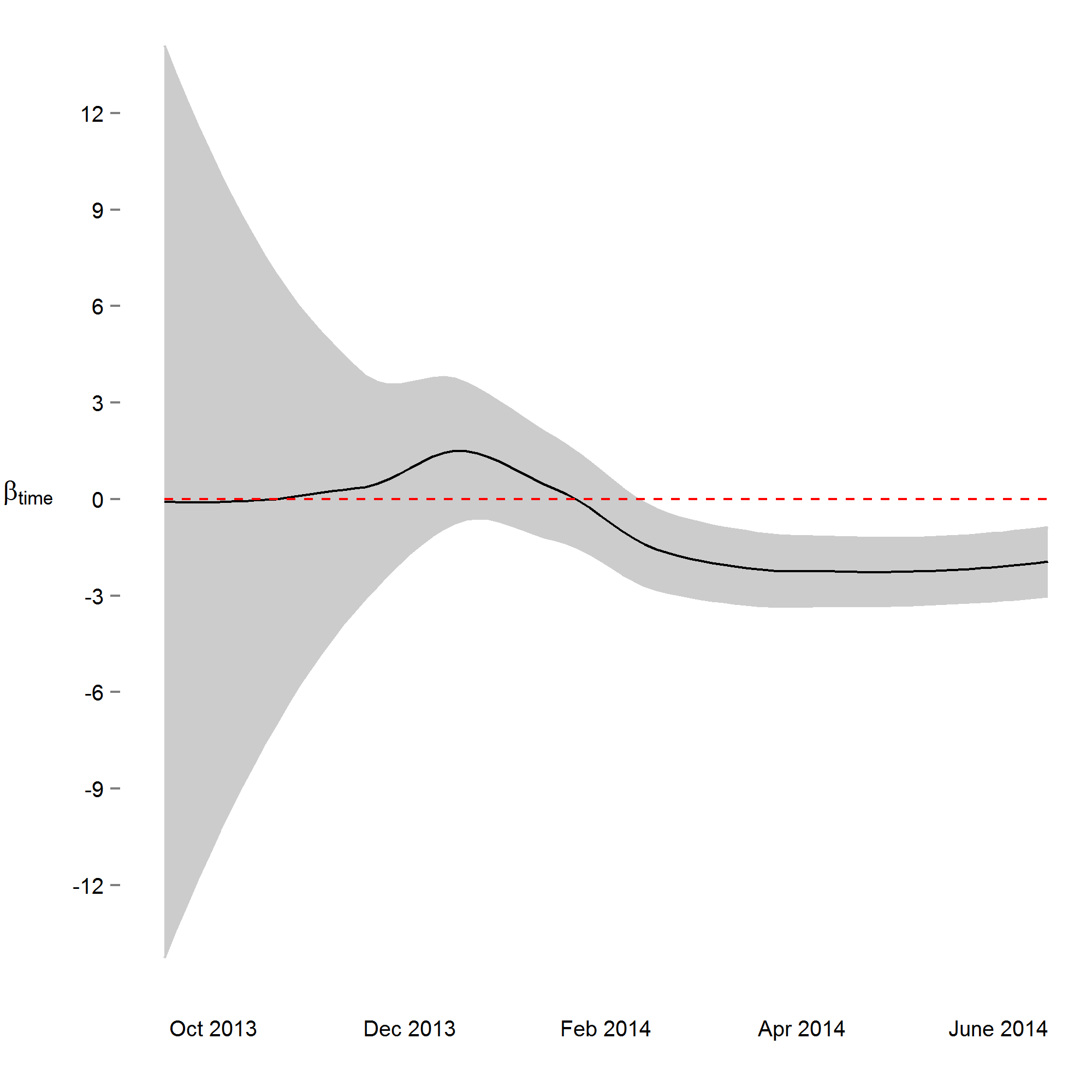

Supplement: Supplemental Information 6 [file peerj-04-2284-s006.png]
