# Supplementary material for: A framework for smartphone-enabled, patient-generated health data analysis
Source: PeerJ. 2016 Aug 2;4:e2284. doi: 10.7717/peerj.2284 (PMC4975026; doi:10.7717/peerj.2284)
Supplement: Supplemental Information 7 — Values are in counts (%) unless otherwise noted. [file peerj-04-2284-s007.docx]

**S1 Table. Study participant demographics at enrollment visit (n=38).** Values are in counts (%) unless otherwise noted.

| Ethnicity | Caucasian | 33 (87) |
| --- | --- | --- |
| Gender | Female | 28 (74) |
| Age | Mean (sd) | 57 (9.44) |
| Education | High school or less | 5 (13) |
|  | College | 18 (47) |
|  | Post-College | 15 (39) |
| Income | Under 50k | 5 (13) |
|  | 50k-99k | 14 (37) |
|  | 100k-149k | 10 (26) |
|  | 150k or more | 9 (24) |
| Phone Use | Did not own smartphone | 8 (21) |
|  | Owned non-iPhone | 11 (29) |
|  | Owned iPhone | 19 (50) |
