# Supplementary material for: A framework for smartphone-enabled, patient-generated health data analysis
Source: PeerJ. 2016 Aug 2;4:e2284. doi: 10.7717/peerj.2284 (PMC4975026; doi:10.7717/peerj.2284)
Supplement: Supplemental Information 8 — Values are in counts (%) unless otherwise noted. * = values in mean (standard deviation). [file peerj-04-2284-s008.docx]

**S2 Table. Study participant self-assessment of health (n=38).** Values are in counts (%) unless otherwise noted. ^*^ = values in mean (standard deviation).

| Health (baseline) | Very good/good | 29 (76) |
| --- | --- | --- |
|  | Average/poor | 9 (24) |
| Health (change) | Increased | 11 (29) |
|  | Same | 25 (66) |
|  | Decreased | 2 (5) |
| Smoking (baseline) | No | 23 (61) |
|  | Yes | 15 (40) |
| Smoking (change) | Increased | 0 (0) |
|  | Same | 23 (61) |
|  | Decreased | 14 (37) |
| Exercise (baseline) | Never | 9 (24) |
|  | Sometimes | 18 (47) |
|  | Often | 11 (29) |
| Exercise (change) | Increased | 11 (29) |
|  | Same | 22 (58) |
|  | Decreased | 4 (11) |
| Doctor Visits^*^ | Baseline | 3.0 (2.6) |
|  | Change | -0.4 (2.7) |
| ER Visits^*^ | Baseline | 0.1 (0.4) |
|  | Change | -0.08 (0.5) |
| Nights in Hospital^*^ | Baseline | 0 (0) |
|  | Change | -0.3 (1.6) |
