# Supplementary material for: A framework for smartphone-enabled, patient-generated health data analysis
Source: PeerJ. 2016 Aug 2;4:e2284. doi: 10.7717/peerj.2284 (PMC4975026; doi:10.7717/peerj.2284)
Supplement: Supplemental Information 9 — Detailed description of statistical modeling. [file peerj-04-2284-s009.docx]

S1 Appendix

**Statistical modeling**

Two approaches were used to assess the change in device readings as a function of time: 1) a multiple N-of-1 approach; and 2) a mixed model combining all individuals.

*Multiple N-of-1 approach*

In this approach, device readings data was analyzed separately on each of the 38 study participants, and then the results were pooled to make an assessment of the overall effect. More explicitly, simple linear regression was first performed on device readings data from each study participant using ‘time’ as the fixed effect variable of interest (quantitative measure; in days since study enrollment) and ‘time of day’ as a categorical fixed effect covariate (rounded to the nearest hour) to account for the known daily trends in blood pressure (e.g., blood pressure is typically lower in the morning and rises during the day). Thus, for each study participant, the following null ($M_{0}$) and alternative ($M_{\text{A}}$) models were constructed:

$$\begin{matrix} M_{0}:Y=\alpha+\sum_{j=1}^{24} \beta_{j}\cdot I\left(\text{hour}=j\right)+\varepsilon\\ M_{\text{A}}:Y=\alpha+\sum_{j=1}^{24} \beta_{j}\cdot I\left(\text{hour}=j\right)+\beta_{time}\left(\text{time}\right)+\varepsilon\end{matrix}$$

where ‘time’ is the time in days of the reading since study enrollment with fixed effect $\beta_{time}$and $H_{0}:\beta_{time}=0$. Furthermore, $Y$is the blood pressure device reading, $\alpha$ is the intercept of the corresponding model, and $I\left(\text{hour}=j\right)$ is an indicator function that is 1 when the hour (rounded) is equal to $j$, with fixed effects $\beta_{j}$ in the corresponding model. Models were constructed for both systolic and diastolic blood pressure, individually.

The estimate of $\beta_{time}$ and p-value related to $H_{0}$ in device readings data from each study participant was recorded. Results were summarized across study participants by assessing the direction of the $\beta_{time}$ estimate and/or related p-value (using a binomial test to test for an enrichment in positive or negative estimates), as well as calculating weighted (by the square root of the number of readings recorded by the study participant) and unweighted average estimates of $\beta_{time}$ and using bootstrapping (1000 iterations) to estimate confidence intervals.

*Mixed model approach*

Alternatively, the ‘time’ trend in device readings data across all study participants simultaneously was examined using a mixed model approach. Similar to the modeling presented above, the primary hypothesis examined if there was an observed change in the device readings data over the course of the study. However, in addition to the ‘time of day’ fixed effect covariate, another condition, the study participant (random effect), needed to be accounted for in order to appropriately assess this hypothesis across study participants in a combined device readings dataset. By doing so, this creates a random intercept model in which device readings data from each study participant are modeled as its own linear trend with a common slope (i.e., the lines for each study participant are parallel). Furthermore, this indirectly accounts for the main effect of individual-specific covariates that may shift each linear trend up or down (remaining parallel) that could otherwise act as confounders, such baseline body mass index or hypertensive medications. We do note, however, that this does not account for interactions between such individual-specific covariates and the trend of ‘time’ (e.g., if baseline BMI moderated the trend of ‘time’; individuals with higher baseline BMI experienced a greater decrease in blood pressure over time than individuals with lower baseline BMI), though typically modeling such interactions is not often performed unless they are the primary hypothesis of interest. As noted in the main text, the general structure of this model is:

$$\mathbf{Y}=\mathbf{X\beta}+\mathbf{Zu}+\mathbf{e}$$

where $\mathbf{Y}$ is a vector of outcomes, $\mathbf{X}$ is a matrix of independent variables with fixed effects $\mathbf{\beta}$, $\mathbf{Z}$ is a matrix indicating the structure of the between subject random effects $\mathbf{u}$ with covariance matrix $\mathbf{G}$, and $\mathbf{e}$ is the random error with covariance matrix $\mathbf{R}$. This model included the fixed effects $\mathbf{\beta}=\left[\beta_{time},\beta_{hour1},\beta_{hour2},\ldots,\beta_{hour24}\right]^{\text{T}}$where, as above, ‘time’ is a quantitative measure representing the days since study enrollment and the effect of each hour represents ‘time of day’ (rounded to the nearest hour) to account for the known daily trends in blood pressure. The primary hypothesis, regardless of the structure of $\mathbf{R}$, assessed $H_{0}:\beta_{time}=0$.

Given the nature of the data (repeated measures taken at unstructured time points), two modeling structures designed to account for the ‘individual’ and ‘time proximity’ between device readings measures were considered: 1) the ‘individual’ random effects $\mathbf{u}$ of the N x n block diagonal identity matrix $\mathbf{Z}$ where N=6,290 is the total number of device readings recorded and n=38 is the number of study participants; and 2) the potential ‘time proximity’ N x N block diagonal covariance matrix $\mathbf{R}$ with elements $\mathbf{\Sigma}_{\text{R}}$. It follows that the covariance matrix $\text{Var}\left(\mathbf{Y}\right)=\mathbf{V}=\mathbf{ZGZ}^{\text{T}}+\mathbf{R}$ is also block diagonal, where each of the n blocks are square with dimensions equal to the number of device readings recorded on a particular study participant.

Three possible covariance structures of $\mathbf{R}$ were modeled, where $\mathbf{\Sigma}_{\text{R}}$ was 1) compound symmetric; 2) first-order autoregressive; or 3) generalized autoregressive for unequally spaced data (i.e. spatial power law). Specifically, if $\rho$ is the correlation between measures on the same study participant, when $\mathbf{\Sigma}_{\text{R}}$ is compound symmetric:

$\mathbf{\Sigma}_{\text{R}}=\sigma^{2}\left(\begin{matrix} 1 & \rho& \rho& \\ \rho& 1 & \rho& \cdots\\ \rho& \rho& 1 & \\ & \vdots& & \ddots\end{matrix}\right)$,

when $\mathbf{\Sigma}_{\text{R}}$ is first-order autoregressive:

$\mathbf{\Sigma}_{\text{R}}=\sigma^{2}\left(\begin{matrix} 1 & \rho& \rho^{2} & \\ \rho& 1 & \rho& \cdots\\ \rho^{2} & \rho& 1 & \\ & \vdots& & \ddots\end{matrix}\right)$,

and when $\mathbf{\Sigma}_{\text{R}}$ is generalized autoregressive:

$$\mathbf{\Sigma}_{\text{R}}=\sigma^{2}\left(\begin{matrix} 1 & \rho^{t_{2}-t_{1}} & \rho^{t_{3}-t_{1}} & \\ \rho^{t_{2}-t_{1}} & 1 & \rho^{t_{3}-t_{2}} & \cdots\\ \rho^{t_{3}-t_{1}} & \rho^{t_{3}-t_{2}} & 1 & \\ & \vdots& & \ddots\end{matrix}\right)$$

where $t_{k}$ is the time of the $k^{\text{th}}$ measurement, and the difference $t_{i}-t_{j}$ is the lag between the $i^{\text{th}}$ and $j^{\text{th}}$ measure. Again, the primary hypothesis, regardless of the structure of $\mathbf{R}$, assessed $H_{0}:\beta_{time}=0$. Models under the alternative hypothesis were compared to each other using AIC and BIC.
